# Short-term clinical efficacy and recorded adverse events of semaglutide combined with dapagliflozin in patients with type 2 diabetes mellitus and chronic kidney disease

**DOI:** 10.1097/MD.0000000000051019

**Published:** 2026-10-09

**Authors:** Cheng Gong, Qiong Luo, Qin Li

**Affiliations:** a Department of Geriatric Medicine, Yangtze River Shipping General Hospital, Hubei, China; b Department of Endocrinology and Metabolism, Yangtze River Shipping General Hospital, Hubei, China.

**Keywords:** chronic kidney disease, dapagliflozin, inflammation, renal function, semaglutide, type 2 diabetes mellitus

## Abstract

Type 2 diabetes mellitus (T2DM) complicated by chronic kidney disease (CKD) is associated with persistent hyperglycemia, progressive renal impairment, systemic inflammation, and an increased risk of adverse clinical outcomes. Sodium–glucose cotransporter 2 inhibitors and glucagon-like peptide-1 receptor agonists have shown metabolic and cardiorenal benefits, but clinical evidence regarding their combined use in patients with T2DM and CKD remains limited. This retrospective observational study included 126 patients with T2DM complicated by CKD who were treated at our hospital between March 2023 and July 2024. Patients were identified through a review of electronic medical records and the application of predefined eligibility criteria. Treatment groups reflected the actual regimen documented in the medical record rather than random allocation. The final analytic cohort comprised 42 patients in each group; the equal group size reflected the eligible records with complete baseline and 8-week follow-up data available in each treatment category during the study period rather than a prospective 1:1:1 enrollment target. The treatment duration was 8 weeks. Glycemic indices, renal function-related parameters, inflammatory markers, clinical response, and recorded adverse events were compared among the 3 groups. After 8 weeks of treatment, fasting plasma glucose, 2-hour postprandial plasma glucose, hemoglobin A1c, blood urea nitrogen, serum creatinine, 24-hour urinary protein, erythrocyte sedimentation rate, interleukin-6, and high-sensitivity C-reactive protein were significantly reduced in all 3 groups compared with baseline. The combination group showed greater short-term reductions in glycemic indices, renal function-related parameters, and inflammatory markers than either monotherapy group. The overall clinical response rate was significantly higher in the combination group than in the dapagliflozin or semaglutide group. In patients with T2DM complicated by CKD, semaglutide combined with dapagliflozin was associated with greater short-term improvements in glycemic control, inflammatory markers, and renal function-related indicators, as well as a higher exploratory clinical response rate, than either monotherapy. No increase in recorded adverse events was detected during 8 weeks of treatment. Because of the retrospective design, modest sample size, and short observation period, these findings should not be interpreted as demonstrating definitive long-term renal benefit or equivalent long-term safety. Further large-scale prospective studies with standardized renal outcomes are required.

## 1. Introduction

Type 2 diabetes mellitus (T2DM) is a chronic metabolic disorder characterized by insulin resistance, progressive pancreatic β-cell dysfunction, and persistent hyperglycemia. Chronic exposure to hyperglycemia contributes to microvascular and macrovascular complications, including diabetic kidney disease, cardiovascular disease, neuropathy, and retinopathy.^[1,2]^ Among these complications, chronic kidney disease (CKD) represents one of the most clinically important conditions because it is associated with progressive decline in renal function, increased cardiovascular risk, impaired quality of life, and higher mortality. In patients with T2DM, CKD commonly manifests as reduced estimated glomerular filtration rate, persistent albuminuria or proteinuria, or both. The coexistence of T2DM and CKD complicates clinical management because reduced renal function may affect drug selection, pharmacokinetics, treatment safety, and long-term prognosis.^[3,4]^

The pathophysiological relationship between T2DM and CKD is complex. Persistent hyperglycemia promotes oxidative stress, activation of the renin–angiotensin–aldosterone system, endothelial dysfunction, glomerular hyperfiltration, advanced glycation end-product accumulation, lipid metabolism disorders, and chronic low-grade inflammation.^[5]^ These abnormalities contribute to glomerular basement membrane thickening, mesangial expansion, tubulointerstitial injury, and progressive renal fibrosis. In addition, CKD itself aggravates insulin resistance and systemic inflammation, thereby further impairing glucose metabolism. This bidirectional interaction increases the difficulty of achieving durable metabolic and renal protection in patients with T2DM complicated by CKD.^[6,7]^

In recent years, sodium–glucose cotransporter 2 inhibitors (SGLT2 inhibitors) and glucagon-like peptide-1 receptor agonists (GLP-1 receptor agonists) have become important therapeutic options for patients with T2DM, particularly those with high cardiovascular or renal risk. Dapagliflozin, a representative SGLT2 inhibitor, reduces renal tubular glucose reabsorption, increases urinary glucose excretion, lowers plasma glucose levels, and improves renal hemodynamics through mechanisms partly independent of insulin secretion.^[8]^ Beyond glycemic control, dapagliflozin may reduce intraglomerular pressure, albuminuria, body weight, and blood pressure, thereby contributing to renal protection. Semaglutide, a long-acting GLP-1 receptor agonist administered once weekly, enhances glucose-dependent insulin secretion, suppresses glucagon release, delays gastric emptying, and improves metabolic regulation. Emerging evidence also suggests that GLP-1 receptor agonists may exert anti-inflammatory, antioxidative, vascular, and renal protective effects.^[9,10]^

Because SGLT2 inhibitors and GLP-1 receptor agonists act through different but potentially complementary mechanisms, their combined use may provide additional metabolic, renal, and anti-inflammatory benefits in patients with T2DM and CKD.^[11,12]^ However, real-world evidence regarding the clinical efficacy and safety of semaglutide combined with dapagliflozin in this patient population remains insufficient. Therefore, this retrospective study evaluated the effects of dapagliflozin, semaglutide, and their combination on glycemic indices, renal function-related parameters, inflammatory markers, clinical response, and adverse events in patients with T2DM complicated by CKD.

## 2. Materials and methods

### 2.1. Study design

This retrospective observational study was approved by the Medical Ethics Committee of Yangtze River Shipping General Hospital and was conducted in accordance with the Declaration of Helsinki. Electronic medical records of patients with T2DM complicated by CKD who were treated at our hospital between March 2023 and July 2024 were reviewed. Patients were screened according to the predefined inclusion and exclusion criteria, including the requirement for complete clinical and laboratory data at baseline and after 8 weeks of treatment. Eligible patients were categorized according to the actual treatment regimen documented in the medical record: dapagliflozin alone, semaglutide alone, or semaglutide combined with dapagliflozin. Treatment assignment was not randomized, and no matching procedure was used. The final analytic cohort comprised 42 patients in each group. The equal group size reflected the number of eligible records with complete baseline and 8-week follow-up data available in each treatment category during the study period rather than a prospectively imposed 1:1:1 enrollment target or sample-size-based allocation. Written informed consent for treatment and use of clinical data was obtained from all patients.

### 2.2. Baseline characteristics

In the dapagliflozin group, there were 28 men and 14 women. The age range was 32 to 69 years, with a mean age of 56.21 ± 9.01 years. Body mass index ranged from 21.45 to 29.14 kg/m^2^, with a mean value of 25.03 ± 3.36 kg/m^2^. The duration of diabetes ranged from 1 to 13 years, with a mean duration of 7.71 ± 3.59 years. CKD stage distribution was as follows: stage 2 in 6 patients, stage 3a in 12 patients, stage 3b in 16 patients, and stage 4 in 8 patients. In the semaglutide group, there were 30 men and 12 women. The age range was 31 to 68 years, with a mean age of 56.13 ± 8.87 years. Body mass index ranged from 21.40 to 29.20 kg/m^2^, with a mean value of 25.16 ± 3.29 kg/m^2^. The duration of diabetes ranged from 1 to 15 years, with a mean duration of 7.65 ± 3.64 years. CKD stage distribution was as follows: stage 2 in 5 patients, stage 3a in 14 patients, stage 3b in 15 patients, and stage 4 in 8 patients. In the combination group, there were 27 men and 15 women. The age range was 32 to 70 years, with a mean age of 56.98 ± 7.69 years. Body mass index ranged from 21.20 to 29.50 kg/m^2^, with a mean value of 25.32 ± 3.29 kg/m^2^. The duration of diabetes ranged from 1 to 14 years, with a mean duration of 7.78 ± 3.84 years. CKD stage distribution was as follows: stage 2 in 6 patients, stage 3a in 14 patients, stage 3b in 17 patients, and stage 4 in 5 patients. No significant differences in baseline characteristics were observed among the 3 groups.

### 2.3. Inclusion and exclusion criteria

The inclusion criteria were as follows: diagnosis of T2DM according to established clinical criteria; CKD defined by persistent renal abnormalities for more than 3 months, including estimated glomerular filtration rate <60 mL/min/1.73 m^2^ or urinary albumin-to-creatinine ratio ≥30 mg/g; age between 30 and 70 years; receipt of dapagliflozin, semaglutide, or their combination according to clinical records; complete clinical and laboratory data before and after treatment; and ability to complete follow-up evaluation. The exclusion criteria were as follows: type 1 diabetes mellitus or other specific types of diabetes; current or planned renal replacement therapy, including hemodialysis, peritoneal dialysis, or kidney transplantation; severe primary cardiac, cerebral, hepatic, or systemic disease; diabetic ketoacidosis within the previous 6 months; recurrent genitourinary tract infection; history of renal surgery; documented allergy or intolerance to dapagliflozin or semaglutide; poor adherence to dietary management or medication; and pregnancy or lactation.

### 2.4. Treatment regimens

Patients in all groups received standard health education, dietary guidance, and routine management of blood pressure, lipids, and other comorbidities according to clinical needs. Patients in the dapagliflozin group received oral dapagliflozin 10 mg once daily in the morning. Patients in the semaglutide group received subcutaneous semaglutide once weekly at an initial dose of 0.25 mg for 4 weeks, followed by dose escalation to 0.5 mg once weekly. Patients in the combination group received dapagliflozin and semaglutide using the same dose regimens as described above. The treatment duration was 8 weeks in all 3 groups. Detailed baseline classes and doses of concomitant medications, including renin-angiotensin system blockers, other antihypertensive agents, lipid-lowering therapy, insulin, and other glucose-lowering drugs, as well as treatment-period medication adjustments, were not consistently extractable from the retrospective records in a standardized form. These variables, therefore, could not be included in the between-group analysis and remain a potential source of residual confounding.

### 2.5. Outcome measures

The primary observation indices included glycemic control, renal function-related parameters, inflammatory markers, clinical response, and recorded adverse events. Glycemic indices included fasting plasma glucose (FPG), 2-hour postprandial plasma glucose (2hPG), and hemoglobin A1c (HbA1c). Renal function-related parameters included blood urea nitrogen (BUN), serum creatinine (SCr), and 24-hour urinary protein excretion (24h-Upro). The estimated glomerular filtration rate (eGFR) was used for CKD diagnosis and baseline staging; however, serial eGFR was not included in the predefined longitudinal outcome dataset available for this retrospective analysis and, therefore, could not be evaluated as a treatment outcome. Accordingly, the renal findings are described as short-term changes in renal function-related indicators rather than as evidence of preserved filtration function or long-term renal benefit. Inflammatory markers included erythrocyte sedimentation rate (ESR), interleukin-6 (IL-6), and high-sensitivity C-reactive protein (hs-CRP). Adverse events recorded during treatment included hypoglycemia, nausea and vomiting, rash, abdominal distension or diarrhea, headache or dizziness, and urinary tract infection. Hypoglycemia was defined as blood glucose ≤3.9 mmol/L.

### 2.6. Definition of clinical response

Clinical response was evaluated according to glycemic control, major symptoms, and 24-hour urinary protein excretion. For this retrospective analysis, “adequate glycemic control” was defined as achievement of the individualized glycemic target documented in the medical record at the 8-week evaluation. Because routine-care targets were individualized and a uniform study-wide numeric cutoff was not available across all records, no single retrospective numeric threshold was imposed. A marked response was defined as adequate glycemic control, disappearance of major symptoms, and a > 50% reduction in 24h-Upro. A response was defined as adequate glycemic control, improvement in major symptoms, and a 10% to 50% reduction in 24h-Upro. No response was defined as failure to meet the above criteria or disease progression. The overall clinical response rate was calculated as follows: Overall clinical response rate = (marked response + response)/total number of patients × 100%. This composite response measure should be interpreted as exploratory because it was not a standardized renal endpoint and included an individualized glycemic-control component.

### 2.7. Statistical analysis

Statistical analysis was performed using SPSS software, version 27.0 (IBM Corporation). Continuous variables were tested for normality using the Kolmogorov–Smirnov test. Normally distributed continuous variables were expressed as mean ± standard deviation. Comparisons among the 3 groups were performed using 1-way analysis of variance, and pairwise comparisons were performed using the least significant difference *t* test. Within-group comparisons before and after treatment were performed using paired-sample *t* tests. Non-normally distributed variables were expressed as median and interquartile range and compared using nonparametric tests. Categorical variables were expressed as number and percentage and compared using the chi-square test. A 2-sided *P* value < .05 was considered statistically significant.

## 3. Results

### 3.1. Comparison of glycemic indices among the 3 groups

Before treatment, FPG, 2hPG, and HbA1c did not differ significantly among the 3 groups. After treatment, all 3 groups showed significant reductions in FPG, 2hPG, and HbA1c compared with baseline. After treatment, the combination group had significantly lower FPG, 2hPG, and HbA1c levels than both monotherapy groups. No significant difference was observed between the dapagliflozin and semaglutide groups (Table 1).

**Table 1:** Comparison of glycemic indices among the 3 groups.

Group	FPG before treatment (mmol/L)	FPG after treatment (mmol/L)	2hPG before treatment (mmol/L)	2hPG after treatment (mmol/L)	HbA1c before treatment (%)	HbA1c after treatment (%)
Dapagliflozin group (n = 42)	9.38 ± 1.88	6.87 ± 1.02*	12.81 ± 1.94	9.05 ± 1.58*	9.26 ± 1.37	7.44 ± 1.20*
Semaglutide group (n = 42)	9.21 ± 1.74	6.69 ± 1.17*	12.94 ± 2.12	8.89 ± 1.49*	9.18 ± 1.29	7.29 ± 1.18*
Combination group (n = 42)	9.43 ± 1.81	5.69 ± 1.10*^,^**^,^***	12.77 ± 2.01	7.32 ± 1.64*^,^**^,^***	9.30 ± 1.42	6.28 ± 1.62*^,^**^,^***
*F* value	0.170	14.069	0.081	15.548	0.085	9.191
*P* value	.844	<.001	.922	<.001	.919	<.001

2hPG = 2-hour postprandial plasma glucose, FPG = fasting plasma glucose, HbA1c = hemoglobin A1c.

* *P* < .05 versus before treatment within the same group.

** P < .05 versus the dapagliflozin group.

*** P < .05 versus the semaglutide group.

### 3.2. Comparison of short-term changes in renal function-related parameters

Before treatment, BUN, SCr, and 24h-Upro did not differ significantly among the 3 groups. After 8 weeks of treatment, all 3 renal function-related indicators were significantly reduced from baseline in each group. The combination group showed significantly lower posttreatment BUN, SCr, and 24h-Upro values than the dapagliflozin and semaglutide groups. No significant difference was found between the 2 monotherapy groups (Table 2). These results represent short-term changes in the measured renal function-related indicators; eGFR was not analyzed longitudinally.

**Table 2:** Comparison of renal function-related parameters among the 3 groups.

Group	BUN before treatment (mmol/L)	BUN after treatment (mmol/L)	SCr before treatment (μmol/L)	SCr after treatment (μmol/L)	24h-Upro before treatment (g)	24h-Upro after treatment (g)
Dapagliflozin group (n = 42)	11.97 ± 2.75	10.55 ± 2.40*	180.25 ± 46.49	164.07 ± 37.04*	1.44 ± 0.46	1.14 ± 0.39*
Semaglutide group (n = 42)	12.25 ± 2.45	10.16 ± 1.95*	182.98 ± 42.76	156.83 ± 32.76*	1.31 ± 0.43	1.12 ± 0.32*
Combination group (n = 42)	12.11 ± 2.69	8.67 ± 1.99*^,^**^,^***	181.34 ± 45.12	129.15 ± 24.22*^,^**^,^***	1.36 ± 0.51	0.89 ± 0.26*^,^**^,^***
*F* value	0.119	9.173	0.039	14.116	0.825	7.550
*P* value	.888	<.001	.961	<.001	.441	.001

24h-Upro = 24-hour urinary protein, BUN = blood urea nitrogen, SCr = serum creatinine.

* *P* < .05 versus before treatment within the same group.

** *P* < .05 versus the dapagliflozin group.

*** *P* < .05 versus the semaglutide group.

### 3.3. Comparison of clinical response

The overall clinical response rate was 80.95% in the dapagliflozin group, 83.33% in the semaglutide group, and 97.62% in the combination group. The overall response rate differed significantly among the 3 groups. The combination group showed a significantly higher overall clinical response rate than both monotherapy groups, whereas no significant difference was observed between the dapagliflozin and semaglutide groups (Table 3).

**Table 3:** Comparison of clinical response among the 3 groups.

Group	Marked response, n (%)	Response, n (%)	No response, n (%)	Overall response rate (%)
Dapagliflozin group (n = 42)	20 (47.62)	14 (33.33)	8 (19.05)	80.95
Semaglutide group (n = 42)	19 (44.19)	16 (38.10)	7 (16.67)	83.33
Combination group (n = 42)	30 (71.43)	11 (23.81)	1 (2.38)	97.62
χ^2^ value	**–**	**–**	**–**	6.167
*P* value	**–**	**–**	**–**	.046

### 3.4. Comparison of inflammatory markers

Before treatment, ESR, IL-6, and hs-CRP did not differ significantly among the 3 groups. After treatment, ESR, IL-6, and hs-CRP decreased significantly in all 3 groups. The combination group had significantly lower posttreatment ESR, IL-6, and hs-CRP levels than the dapagliflozin and semaglutide groups. In addition, posttreatment IL-6 and hs-CRP levels were significantly lower in the semaglutide group than in the dapagliflozin group (Table 4).

**Table 4:** Comparison of inflammatory markers among the 3 groups.

Group	ESR before treatment (mm/h)	ESR after treatment (mm/h)	IL-6 before treatment (ng/L)	IL-6 after treatment (ng/L)	hs-CRP before treatment (mg/L)	hs-CRP after treatment (mg/L)
Dapagliflozin group (n = 42)	29.43 ± 3.29	17.83 ± 2.76*	106.64 ± 22.76	83.27 ± 17.70*	6.82 ± 0.83	5.56 ± 0.69*
Semaglutide group (n = 42)	30.64 ± 3.75	17.35 ± 2.59*	104.98 ± 23.28	75.48 ± 16.98*^,^**	6.59 ± 0.79	4.85 ± 0.58*^,^**
Combination group (n = 42)	29.89 ± 3.16	15.43 ± 2.32*^,^**^,^***	109.70 ± 20.69	48.98 ± 11.46*^,^**^,^***	6.39 ± 0.88	2.27 ± 0.63*^,^**^,^***
*F* value	1.348	10.311	0.485	55.548	2.795	312.284
*P* value	.264	<.001	.617	<0.001	.065	<.001

ESR = erythrocyte sedimentation rate, IL-6 = interleukin-6, hs-CRP = high-sensitivity C-reactive protein.

* *P* < .05 versus before treatment within the same group.

** *P* < .05 versus the dapagliflozin group.

*** *P* < .05 versus the semaglutide group.

### 3.5. Comparison of recorded adverse events during the 8-week observation period

No statistically significant between-group differences were detected in the recorded incidence of hypoglycemia, nausea and vomiting, rash, abdominal distension or diarrhea, headache or dizziness, or urinary tract infection during the 8-week observation period (Table 5). Given the retrospective ascertainment, modest sample size, and short follow-up, this finding is interpreted as no detected increase in recorded adverse events rather than evidence of equivalent or long-term safety.

**Table 5:** Comparison of adverse events among the 3 groups.

Group	Hypoglycemia, n (%)	Nausea and vomiting, n (%)	Rash, n (%)	Abdominal distension or diarrhea, n (%)	Headache or dizziness, n (%)	Urinary tract infection, n (%)
Dapagliflozin group (n = 42)	3 (7.14)	3 (7.14)	1 (2.38)	3 (7.14)	1 (2.38)	1 (2.38)
Semaglutide group (n = 42)	3 (7.14)	4 (9.52)	1 (2.38)	3 (5.45)	1 (2.38)	0 (0.00)
Combination group (n = 42)	1 (2.38)	2 (4.76)	0 (0.00)	2 (4.76)	0 (0.00)	1 (2.38)
χ^2^ value	1.210	0.718	1.016	0.267	1.016	1.016
*P* value	.546	.698	.602	.875	.602	.602

## 4. Discussion

This retrospective observational study evaluated 8-week clinical changes associated with dapagliflozin, semaglutide, and their combination in patients with T2DM complicated by CKD. All 3 regimens were associated with significant short-term reductions in glycemic indices, renal function-related indicators, and inflammatory markers. The combination group showed greater reductions in FPG, 2hPG, HbA1c, BUN, SCr, 24h-Upro, ESR, IL-6, and hs-CRP than either monotherapy group and had the highest exploratory overall clinical response rate. No statistically significant between-group increase in recorded adverse events was detected during the 8-week observation period. These findings should be interpreted within the limits of the retrospective design and short follow-up and do not establish definitive long-term renal benefit or equivalent safety.

The improvement in glycemic control observed in all groups is consistent with the pharmacological characteristics of dapagliflozin and semaglutide. Dapagliflozin lowers plasma glucose by inhibiting SGLT2-mediated glucose reabsorption in the proximal renal tubules, thereby increasing urinary glucose excretion. This mechanism is largely independent of insulin secretion and may remain clinically useful in patients with insulin resistance.^[13]^ Semaglutide improves glycemic control through glucose-dependent stimulation of insulin secretion, inhibition of glucagon secretion, delayed gastric emptying, and appetite regulation. Because these 2 agents target different metabolic pathways, their combined use may provide complementary glucose-lowering effects.^[12,14]^ The present study showed that posttreatment FPG, 2hPG, and HbA1c were significantly lower in the combination group than in either monotherapy group, supporting the potential additive metabolic benefit of the combined regimen.

Renal function-related indicators showed short-term improvement after treatment, particularly in the combination group. The reductions in BUN, SCr, and 24h-Upro indicate more favorable 8-week changes in these measured indicators with combined semaglutide and dapagliflozin than with either agent alone; however, they should not be interpreted as evidence of sustained preservation of glomerular filtration or long-term renal protection. Dapagliflozin may reduce intraglomerular hypertension through restoration of tubuloglomerular feedback, leading to lower albuminuria or proteinuria.^[15]^ Semaglutide may influence renal risk through metabolic improvement, weight reduction, natriuresis, blood pressure reduction, and attenuation of inflammatory and oxidative pathways.^[16,17]^ These mechanisms provide biological plausibility for the observed short-term changes, but the present study did not include longitudinal eGFR or urinary albumin-to-creatinine ratio and was not designed to evaluate hard renal outcomes.

Inflammation is an important contributor to the development and progression of both T2DM and CKD. Chronic hyperglycemia and renal dysfunction can induce oxidative stress, endothelial injury, and activation of proinflammatory signaling pathways. Elevated inflammatory markers, including IL-6 and hs-CRP, are associated with vascular injury, renal fibrosis, cardiovascular risk, and adverse outcomes in patients with CKD.^[18,19]^ In the present study, inflammatory markers decreased significantly in all treatment groups, with the greatest reductions observed in the combination group. These findings suggest that combined therapy may have a stronger association with inflammatory suppression than monotherapy. The semaglutide group also showed lower posttreatment IL-6 and hs-CRP levels than the dapagliflozin group, which may be related to the anti-inflammatory effects of GLP-1 receptor activation.^[20]^ However, because this was a retrospective study, causal inference regarding anti-inflammatory mechanisms should be made cautiously. The overall clinical response rate was significantly higher in the combination group than in either monotherapy group. This finding is consistent with the greater improvements in glucose metabolism, renal function-related indicators, and inflammatory markers observed in the combination group. However, the clinical response definition used in this study included symptom improvement and 24h-Upro reduction, and therefore should be interpreted as an exploratory composite response measure rather than a standardized renal outcome. In future studies, more internationally recognized endpoints, such as changes in estimated glomerular filtration rate, urinary albumin-to-creatinine ratio, sustained decline in estimated glomerular filtration rate, progression to kidney failure, cardiovascular events, and all-cause mortality, should be used to strengthen clinical interpretation.

Safety is an important consideration when combining glucose-lowering agents in patients with CKD. In this study, no statistically significant between-group differences were detected in the recorded incidence of hypoglycemia, gastrointestinal symptoms, rash, abdominal distension or diarrhea, headache or dizziness, or urinary tract infection during 8 weeks of treatment. This finding indicates that an increase in these recorded adverse events was not detected in this cohort; it does not demonstrate safety equivalence, exclude uncommon adverse events, or establish longer-term safety. Patients with CKD may be more vulnerable to volume depletion, gastrointestinal intolerance, and genitourinary infection.^[21,22]^ Therefore, careful patient selection, renal function monitoring, hydration assessment, and individualized treatment remain necessary in clinical practice.

This study has several limitations. First, the retrospective design limited causal inference and introduced potential selection bias and confounding by indication. Second, the sample size was modest, and all patients were treated at a single center, which may reduce generalizability and limit the ability to detect uncommon adverse events. Third, the treatment duration was only 8 weeks, which was insufficient to evaluate sustained renal outcomes, cardiovascular events, or long-term safety. Fourth, although eGFR was used for CKD diagnosis and baseline staging, serial eGFR was not included in the predefined longitudinal outcome dataset; urinary albumin-to-creatinine ratio was also not analyzed. Therefore, the observed changes in BUN, SCr, and 24h-Upro should be interpreted as short-term changes in renal function-related indicators rather than definitive renal benefit. Fifth, detailed baseline concomitant medication classes, doses, and treatment-period adjustments were not consistently extractable in a standardized form, preventing adjustment for potential effects of renin-angiotensin system blockers, other antihypertensive agents, lipid-lowering drugs, insulin, and other glucose-lowering therapies. Residual confounding from medication changes, dietary sodium intake, blood pressure control, lipid management, baseline albuminuria, and CKD etiology, therefore, cannot be excluded. Sixth, the exploratory clinical response measure included achievement of an individualized glycemic target documented in routine care rather than a uniform study-wide numeric threshold, which limits standardization and comparability. Future multicenter prospective studies with larger samples, longer follow-up, standardized eGFR- and albuminuria-based renal endpoints, prespecified safety surveillance, and adjustment for concomitant therapies are warranted.

## 5. Conclusion

In patients with T2DM complicated by CKD, semaglutide combined with dapagliflozin was associated with greater short-term improvements in glycemic indices, inflammatory markers, and renal function-related indicators, as well as a higher exploratory overall clinical response rate, compared with either monotherapy over 8 weeks. No increase in recorded adverse events was detected during the observation period. Owing to the retrospective design, modest sample size, incomplete control of concomitant therapies, absence of longitudinal eGFR assessment, and short follow-up, these findings should not be interpreted as demonstrating definitive long-term renal benefit or equivalent long-term safety. Prospective multicenter studies using standardized renal and safety endpoints are needed to validate these observations.

## Author contributions

**Conceptualization:** Cheng Gong, Qiong Luo, Qin Li.

**Data curation:** Cheng Gong, Qiong Luo, Qin Li.

**Formal analysis:** Cheng Gong, Qiong Luo, Qin Li.

**Funding acquisition:** Qin Li.

**Investigation:** Qin Li.

**Writing – original draft:** Qin Li.

**Writing – review & editing:** Qin Li.

## References

[1] de Boer IH Khunti K Sadusky T. Diabetes management in chronic kidney disease: a consensus report by the American Diabetes Association (ADA) and Kidney Disease: Improving Global Outcomes (KDIGO). Diabetes Care. 2022;45:3075–90. 10.2337/dci22-0027 PMC9870667 36189689

[2] de Boer IH Khunti K Sadusky T. Diabetes management in chronic kidney disease: a consensus report by the American Diabetes Association (ADA) and Kidney Disease: Improving Global Outcomes (KDIGO). Kidney Int. 2022;102:974–89. 10.1016/j.kint.2022.08.012 36202661

[3] Irvania AN Tsauri GAA Yantisetiasti A Sofiatin Y. Dapagliflozin improves hemoglobin and anemia in chronic kidney disease: a systematic review and meta-analysis. Ren Fail. 2026;48:2636407. 10.1080/0886022X.2026.2636407 PMC13107988 42021630

[4] Navaneethan SD Zoungas S Caramori ML. Diabetes management in chronic kidney disease: synopsis of the KDIGO 2022 clinical practice guideline update. Ann Intern Med. 2023;176:381–7. 10.7326/M22-2904 36623286

[5] Zhang H Wang K Zhao H. Diabetic kidney disease: from pathogenesis to multimodal therapy-current evidence and future directions. Front Med (Lausanne). 2025;12:1631053. 10.3389/fmed.2025.1631053 PMC12370670 40861214

[6] Tang G Li S Zhang C Chen H Wang N Feng Y. Clinical efficacies, underlying mechanisms and molecular targets of Chinese medicines for diabetic nephropathy treatment and management. Acta Pharm Sin B. 2021;11:2749–67. 10.1016/j.apsb.2020.12.020 PMC8463270 34589395

[7] Martos-Guillami N Vergara A Llorens-Cebrià C. SGLT2i and GLP1-RA exert additive cardiorenal protection with a RAS blocker in uninephrectomized db/db mice. Front Pharmacol. 2024;15:1415879. 10.3389/fphar.2024.1415879 PMC11491409 39434906

[8] Jhund PS Solomon SD Docherty KF. Efficacy of dapagliflozin on renal function and outcomes in patients with heart failure with reduced ejection fraction: results of DAPA-HF. Circulation. 2021;143:298–309. 10.1161/CIRCULATIONAHA.120.050391 PMC7834909 33040613

[9] Perkovic V Tuttle KR Rossing P. Effects of semaglutide on chronic kidney disease in patients with type 2 diabetes. N Engl J Med. 2024;391:109–21. 10.1056/NEJMoa2403347 38785209

[10] Badve SV Bilal A Lee MMY. Effects of GLP-1 receptor agonists on kidney and cardiovascular disease outcomes: a meta-analysis of randomised controlled trials. Lancet Diabetes Endocrinol. 2025;13:15–28. 10.1016/S2213-8587(24)00271-7 39608381

[11] Apperloo EM Neuen BL Fletcher RA. Efficacy and safety of SGLT2 inhibitors with and without glucagon-like peptide 1 receptor agonists: a SMART-C collaborative meta-analysis of randomised controlled trials. Lancet Diabetes Endocrinol. 2024;12:545–57. 10.1016/S2213-8587(24)00155-4 38991584

[12] Neuen BL Fletcher RA Heath L. Cardiovascular, kidney, and safety outcomes with GLP-1 receptor agonists alone and in combination with SGLT2 inhibitors in type 2 diabetes: a systematic review and meta-analysis. Circulation. 2024;150:1781–90. 10.1161/CIRCULATIONAHA.124.071689 39210781

[13] Upadhyay A. SGLT2 inhibitors and kidney protection: mechanisms beyond tubuloglomerular feedback. Kidney360. 2024;5:771–82. 10.34067/KID.0000000000000425 PMC11146657 38523127

[14] The Nuffield Department of Population Health Renal Studies Group, the SGLT2 inhibitor Meta-Analysis Cardio-Renal Trialists’ Consortium. Impact of diabetes on the effects of sodium glucose co-transporter-2 inhibitors on kidney outcomes: collaborative meta-analysis of large placebo-controlled trials. Lancet. 2022;400:1788–801. 10.1016/S0140-6736(22)02074-8 PMC7613836 36351458

[15] Zhu X Wang X Zhang P. Efficacy and safety of novel antidiabetic drugs in patients with type 2 diabetes and chronic kidney disease: a network meta-analysis. Front Endocrinol (Lausanne). 2026;17:1750615. 10.3389/fendo.2026.1750615 PMC13076170 41987895

[16] Alkhezi O Alfehaid L Alanezi A Alotaibi M Alshaya O. Real-world use of semaglutide in patients with type 2 diabetes and end-stage renal disease: a multicenter retrospective cohort study. Front Endocrinol (Lausanne). 2026;17:1805015. 10.3389/fendo.2026.1805015 PMC13061728 41970978

[17] Mann JFE Rossing P Bakris G. Effects of semaglutide with and without concomitant SGLT2 inhibitor use in participants with type 2 diabetes and chronic kidney disease in the FLOW trial. Nat Med. 2024;30:2849–56. 10.1038/s41591-024-03133-0 PMC11485243 38914124

[18] Alicic RZ Rooney MT Tuttle KR. Diabetic kidney disease: challenges, progress, and possibilities. Clin J Am Soc Nephrol. 2017;12:2032–45. 10.2215/CJN.11491116 PMC5718284 28522654

[19] Mosenzon O Capehorn MS De Remigis A Rasmussen S Weimers P Rosenstock J. Impact of semaglutide on high-sensitivity C-reactive protein: exploratory patient-level analyses of SUSTAIN and PIONEER randomized clinical trials. Cardiovasc Diabetol. 2022;21:172. 10.1186/s12933-022-01585-7 PMC9440529 36056351

[20] Alharbi SH. Anti-inflammatory role of glucagon-like peptide 1 receptor agonists and its clinical implications. Ther Adv Endocrinol Metab. 2024;15:20420188231222367. 10.1177/20420188231222367 PMC10823863 38288136

[21] Chiang YC Dai DF Chiu YW Lin HY. Sodium-glucose cotransporter-2 inhibitors and acute kidney injury risk: a systematic review and meta-analysis of randomized trials. Kidney Med. 2026;8:101248. 10.1016/j.xkme.2026.101248 PMC12937176 41767694

[22] Menne J Dumann E Haller H Schmidt BMW. Acute kidney injury and adverse renal events in patients receiving SGLT2-inhibitors: a systematic review and meta-analysis. PLoS Med. 2019;16:e1002983. 10.1371/journal.pmed.1002983 PMC6901179 31815931

